# A New Variant of Type I Congenital Ulna Deficiency With the Normal Thumb, Webspace, Hand, and Elbow

**DOI:** 10.7759/cureus.12261

**Published:** 2020-12-24

**Authors:** Terrence Jose Jerome, Ramesh Prabu, Thirumagal Kuppusamy Terrence

**Affiliations:** 1 Orthopaedics, Hand and Reconstructive Microsurgery, Olympia Hospital and Research Centre, Trichy, IND; 2 Orthopaedics and Traumatology, K.A.P. Vishwanatham Medical College Hospital, Mahatma Gandhi Memorial Hospital, Trichy, IND; 3 Reproductive Medicine/Obstetrics and Gynecology, Olympia Hospital and Research Centre, Trichy, IND

**Keywords:** a new variant, type i congenital ulna deficiency, normal thumb, webspace, hand and elbow

## Abstract

Unilateral congenital ulna deficiency of wrist and forearm is rare. It is associated with cartilaginous ulnar anlage, absence of ulnar digits, carpus, partially or completely absent ulna, radiohumeral synostosis, syndactyly, and thumb abnormalities. Various classifications have described this presentation. We report a new variant of type I congenital ulnar deficiency in wrist and forearm with a normal thumb, first webspace, hand, wrist, and elbow in an 18-year-old girl.

## Introduction

Congenital ulna deficiency is associated with a broad spectrum of musculoskeletal anomalies in the upper and lower limbs [1]. Careful physical evaluation supplemented with radiographs is essential for management [2]. There have been many classifications based on the elbow, forearm, carpal and finger deficiencies, thumb and first web anomalies [1-4]. Type I is a mild form of deficiency associated with normal first webspace and thumb, and absence of ulnar digits and carpus, carpal bone fusion, and syndactyly in hand. Also, it has ulnar deficiency with cartilaginous anlage, radial head dislocation, restriction of elbow flexion/extension, forearm pronation/supination, absent cubital web [1].

We report a new variant of type I congenital ulna deficiency with normal hand digits, thumb, first webspace, carpus, and elbow with ulnar hypoplasia in an 18-year-old girl with a normal function of the upper limb.

## Case presentation

**Figure 1:**
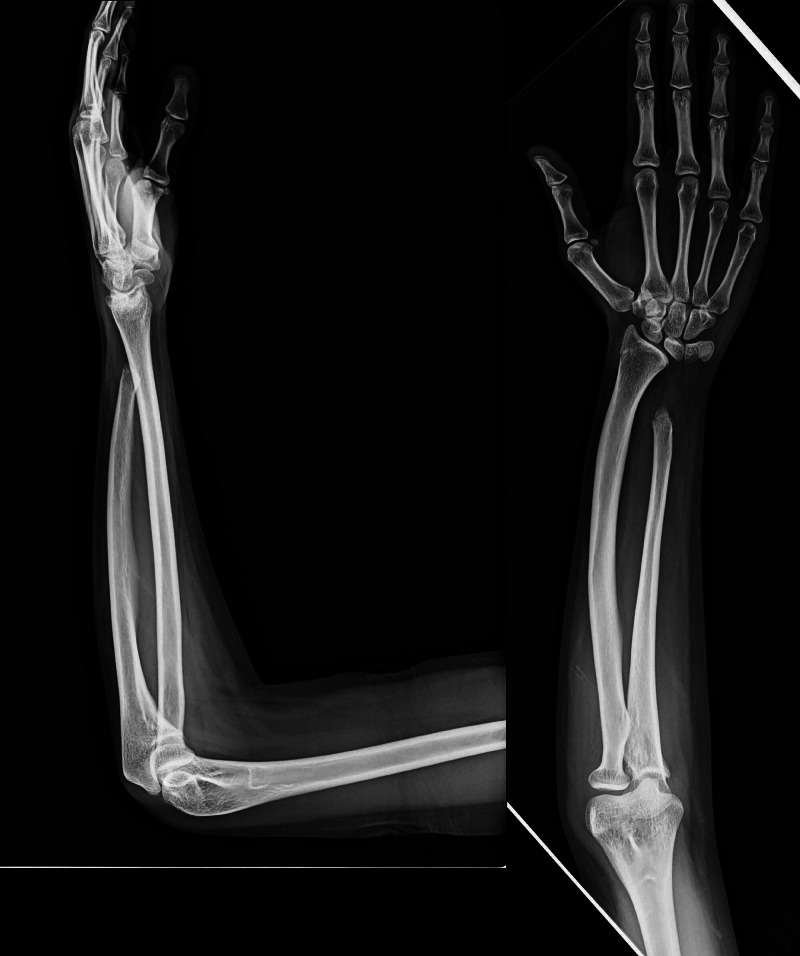
Radiographs show new variant type I congenital ulna deficiency right forearm and wrist Radiographs show a new variant type I congenital ulna deficiency right forearm and wrist of an 18-year old girl at the time of skeletal maturity. The posteroanterior and lateral views show ulna hypoplasia and radius bowing with normal hand, five digits, and normal elbow joint.

We present the case of a girl who initially reported to us with a shortening right upper limb. She was 13 years old then. Clinical examination revealed bowing of the radius, absence of distal ulna, and a 1.5 cm true shortening of the right forearm. Also, fibrous tissue filled the ulna deficient space along with the extensor tendons in the ulnar aspect of the distal forearm. The shoulder, elbow, and wrist movements were normal. Hand, thumb, and first webspace were also normal with all five digits. Radiographs of the forearm revealed congenital type I ulnar deficiency with the increased ulnar slope of the distal radius and 10 degrees bowing. Since she had normal activities, we counseled her about the prognosis of the deformity and advised her to follow-up till skeletal maturity. At 18 years, she visited our clinic with occasional pain in the elbow and wrist. Examination revealed 25 degrees of radial bowing and 2.5 cm true shortening in the right forearm. There was no proximal and distal radioulnar instability. There was no radial head dislocation (Figure 1).

**Figure 2:**
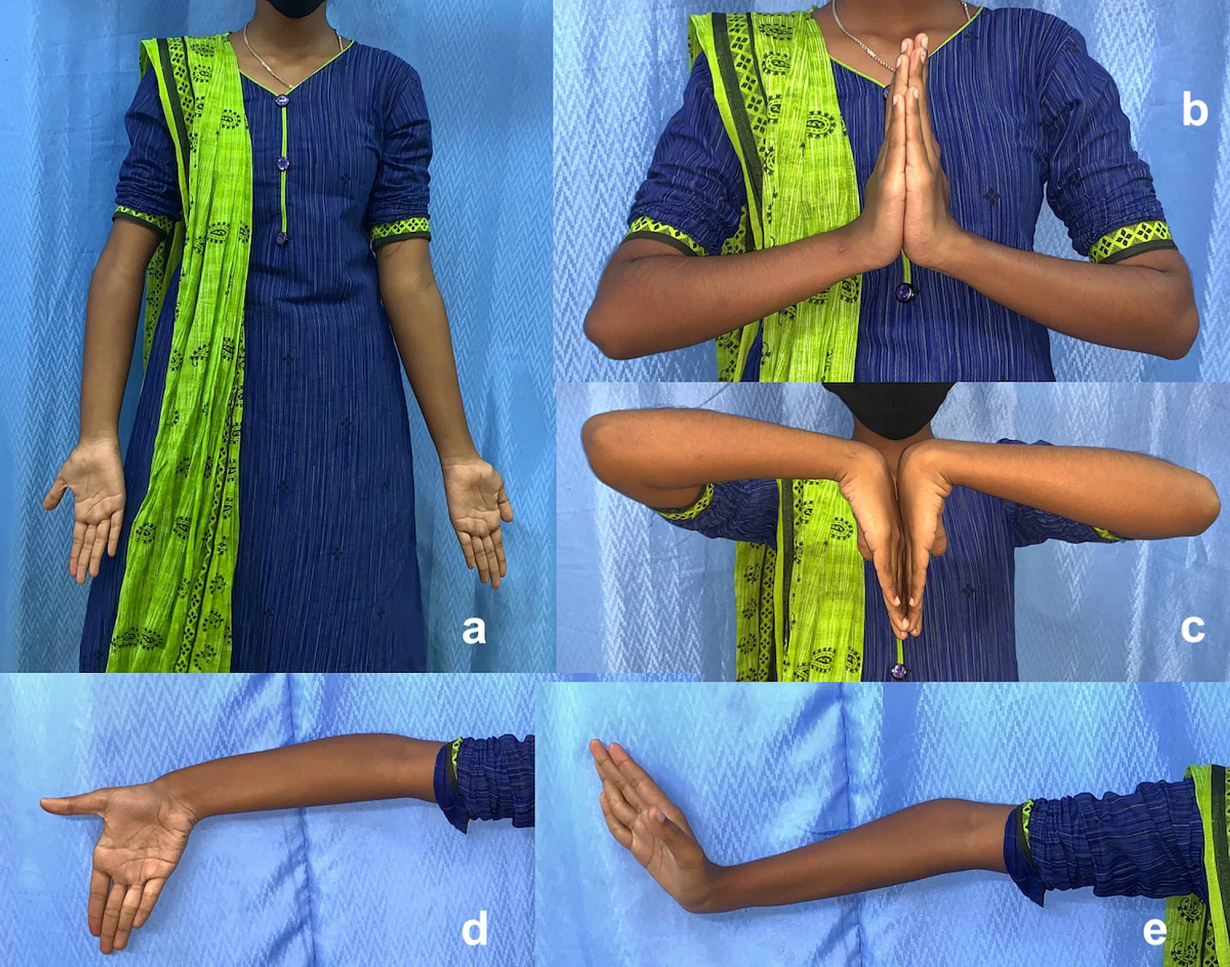
Clinical appearance Clinical appearance of the girl with the new variant of type I congenital ulna deficiency right forearm and wrist shows radial bowing, shortening, and normal hand. (a-e) The elbow, wrist, and hand functions are normal.

She retained her full range of movements in the shoulder, elbow, wrist, and hand. Also, she managed these years with the shortening and occasional pain on strenuous activities (Figure 2).

## Discussion

Kümmel reported a classification based on the morphological findings of the elbow: normal or near-normal humeroradial joint, humeroradial synostosis, and radial head dislocation [5]. Ogino and Kato classified the hand abnormalities based on the missing ulnar rays and ulnar defect: hypoplasia, partial defect, and total defect of the ulna [6]. It did not include the thumb anomalies, which is seen in 70% of congenital ulnar deficiency [7-11]. Broudy and Smith reported 100% hand abnormalities in congenital ulna deficiency and did not have classification-based treatment [7]. Also, they noted elbow dislocation (65%), the complete absence of ulna (40%), hypoplastic ulna proximal and distal (40%), and humerus ankylosis (20%). None of the patients had normal elbow. 

Swanson et al. reported 68% hand abnormalities, 53.4% humeroradial synostosis, the complete absence of ulna (24%), and congenital wrist amputation (5%) in ulnar deficiency [1]. They have described hypoplasia or partial defect of the ulna as type I, total defect of the ulna as type II, partial or total defect with humeroradial synostosis as type III, and partial or total defect of the ulna associated with congenital wrist amputation as type IV. None of the patients had normal elbow, carpus and hand with five digits. 

Based on our case, we propose a new variant of type I with a normal hand with five digits, a normal elbow, and a full range of function in the entire forearm (Table 1).

**Table 1:** Revised type I classification of ulnar deficiency based on elbow and forearm anomalies

Characteristics	Range of motion
Elbow	Normal
Normal humeroradial joint	
No radial head dislocation	
Radius	
Bowing	Normal
Ulna length	
Ulnar hypoplasia	
Shortening 2.5 cms	Normal
Wrist	
Cartilaginous ulnar “anlage”	
Normal carpus	
Hand	Normal
Five digits	
Normal thumb and first webspace	
Absent syndactyly	
Other upper extremity characteristics (None)	Normal

Our case had normal movements in the upper limb with occasional pain during her activities. This new variant type I ulnar deficiency does not require any treatment. This type has to be followed until skeletal maturity. Observation of the case with no treatment and available evidence are the limitations of this report. Also, we are unclear and not sure that this congenital ulnar deficiency shall never require any treatment during or in the course of observation. 

## Conclusions

We report a new variant of type I congenital ulnar deficiency wrist and forearm with normal hand, five digits, and elbow, with distal ulnar hypoplasia. The functions are normal in this type of deficiency and require no treatment. Follow-up till the skeletal maturity is mandated. Given this single case with limited evidence, we are also uncertain of the indication for surgical intervention.
